# Use of a Zwitterionic Surfactant to Improve the Biofunctional Properties of Wool Dyed with an Onion (*Allium cepa* L.) Skin Extract

**DOI:** 10.3390/antiox9111055

**Published:** 2020-10-28

**Authors:** Chiara Puri, Lucia Pucciarini, Matteo Tiecco, Virginia Brighenti, Claudia Volpi, Marco Gargaro, Raimondo Germani, Federica Pellati, Roccaldo Sardella, Catia Clementi

**Affiliations:** 1 Department of Chemistry Biology and Biotechnology, University of Perugia, Via Elce di Sotto 8, 06123 Perugia, Italy; puric.biotec@gmail.com (C.P.); matteotiecco@gmail.com (M.T.); raimondo.germani@unipg.it (R.G.); 2 Department of Pharmaceutical Sciences, University of Perugia, Via Fabretti 48, 06123 Perugia, Italy; lucia.pucciarini@hotmail.it; 3 Department of Life Sciences, University of Modena and Reggio Emilia, Via G. Campi 103, 41125 Modena, Italy; virginia.brighenti@unimore.it (V.B.); federica.pellati@unimore.it (F.P.); 4 Department of Experimental Medicine, University of Perugia, P.le Severi, 06132 Perugia, Italy; claudia.volpi@unipg.it (C.V.); marco.gargaro@unipg.it (M.G.); 5 Center for Perinatal and Reproductive Medicine, University of Perugia, Santa Maria della Misericordia/University Hospital, 06132 Perugia, Italy

**Keywords:** onion, flavonoids, quercetin, waste, surfactant, antioxidant activity, biofunctional textiles

## Abstract

To improve the loadability and antioxidant properties of wool impregnated with onion skin extract, the introduction of SB3-14 surfactant in the dyeing process was evaluated. A preliminary investigation on the surfactant–quercetin interaction indicated that the optimal conditions for dye solubility, stability, and surfactant affinity require double-distilled water (pH = 5.5) as a medium and SB3-14 in a concentration above the c.m.c. (2.5 × 10^−3^ M). The absorption profile of textiles showed the flavonoid absorption band (390 nm) and a bathochromic feature (510 nm), suggesting flavonoid aggregates. The higher absorbance for the sample dyed with SB3-14 indicated greater dye uptake, which was further confirmed by HPLC analysis. The Folin–Ciocalteu method was applied to evaluate the total phenol content (TPC) released from the treated wool, while the assays FRAP, DPPH, ABTS, and ORAC were applied to evaluate the corresponding total antioxidant activity (TAC). Higher TPCs (about 20%) and TACs (5–55%) were measured with SB3-14, highlighting textiles with improved biofunctional properties. Spectrophotometric analyses were also performed with an artificial sweat. The potential cytotoxic effect of SB3-14 in both monomeric and aggregated forms, cell viability, and induction of apoptosis were evaluated in RAW 264.7 cells. These analyses revealed that SB3-14 is safe at concentrations below the c.m.c.

## 1. Introduction

The exploitation of plant extracts as a raw source of colored bioactive compounds has gained increasing attraction in the last few years for the concrete possibility of performing a sustainable production of colored textiles with specific health-promoting properties on human skin [1,2,3]. These innovative textile products are mainly referred to as biofunctional textiles or cosmetotextiles. Different technologies and methodologies have been developed so far to enhance the loadability of these materials by bioactive compounds, with the objective of promoting their slow and continuous release on the surface of the human body through the direct contact with the skin [4,5,6]. Accordingly, both highly advanced technological methods and rather traditional practices have been proposed. In this scenario, natural dyes are particularly appreciated, due to their low toxicity, high biocompatibility, and biodegradability, along with minimum environmental and ecological impact. 

In addition to the most common coloring function, natural dyes have also been well-known for a long time for a number of other interesting properties, including antioxidant activity, UV solar protection ability, as well as for their antibacterial and insect repellent action. Very interestingly, all these properties are entirely retained when the bioactive molecules are transferred from the plant extract to a textile substrate [7]. 

In the multitude of compounds usually constituting the bioactive fractions of the extracted phytocomplex, flavonoids are recognized among the most interesting species as they exhibit potent antioxidant and anti-inflammatory effects [8]. Indeed, due to their ability to scavenge free oxygen radicals, flavonoids may help reduce aging-related processes, protect human skin from the lipoperoxidation caused by UV radiation, and inhibit the onset of degenerative diseases, such as atherosclerosis and cancer. Therefore, many studies have been carried out to explore the multifunctional properties of textiles dyed with pure flavonoid dyes or flavonoid-based plant extracts [9,10,11]. 

While the early studies on biofunctional textiles were performed rather empirically, during the last decade the way to investigate these matrices has markedly evolved giving solid scientific fundamentals to the experimental outcomes. Accordingly, in a recent work [12], the application of a multi-analytical approach based on advanced chromatographic, cytofluorimetric, and spectroscopic studies allowed us to demonstrate that wool yarns impregnated with the phenol pool extracted from dry yellow onion (“Dorata di Parma” cultivar) peel exhibit a high potential in protecting human skin against lipid peroxidation following UV radiation, without cellular toxic effects. This biomass was rationally selected for our studies, as onion is one of the most worldwide diffused horticultural crops with a current production of several million tons around the world [12]. Therefore, its related by-products are widespread and easily available. Furthermore, onion skin waste is easily handled because of its low weight and dryness state. More to the point, onion skin extracts have a long history in the framework of the traditional dyeing processes, making the use of this biomass a valuable bridge between tradition and innovation. 

The performance and durability of biofunctional textiles can be improved by developing strategies aimed at increasing the loadability of active principles on the fiber substrate without compromising the release behavior. This goal can be conveniently achieved either through specific technologies and methodologies (such as microencapsulation, surface textile modification, etc.) or, alternatively, promoting the absorption of the active agent(s) through the formation of additional weak chemical bonds with a textile matrix [4,5,13]. Following the latter strategy, in this paper we describe the effect of the addition of a zwitterionic surfactant in the dyeing step, as a valuable tool to promote the enhancement of the wool fiber loadability. 

Surfactants are extensively used in the textile industry because they can play an important role in all water treatments involving the vehiculation of chemicals into textile fibers. Indeed, by lowering the surface tension of aqueous solution, they improve the wettability of fibers, thus providing a deeper and more homogeneous penetration of the vehiculated molecules within them. They are also used to favor the uniform dispersion of chemical compounds in water. Therefore, it is evident that surfactants can play a fundamental role in the dyeing process as dispersing, wetting, and leveling agents enabling a uniform depth coloration of textiles. While surfactants are massively used in the dyeing industry involving synthetic dyes, their application with plant colorants is still poorly explored. The few reports concerning the interaction of natural dye extracts with surfactants are mainly focused on spectral, thermodynamic, and surface tension investigations [14,15]. However, a recent paper reports the influence of ionic surfactants in enhancing the dye uptake and antimicrobial activity of silk and cotton yarns dyed with natural compounds [16]. 

**Figure 1:**
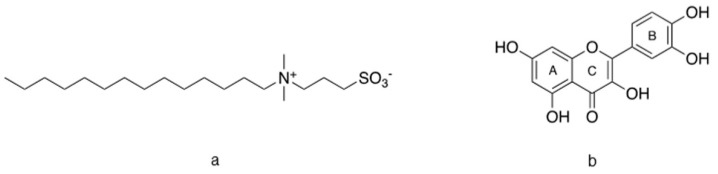
Molecular structure of (**a**) 3-(N,N-dimethyltetradecylammonio)propane-1-sulfonate (SB3-14), and (**b**) quercetin.

With the aim of improving the loadability and the biofunctional properties of wool yarn dyed with a dry onion skin aqueous extract, the role of a zwitterionic sulfobetaine-based surfactant, 3-(N,N-dimethyltetradecylammonio)propane-1-sulfonate, labelled as SB3-14 (Figure 1a), in the dyeing process was evaluated in this work. Zwitterionic surfactants are very popular in pharmaceutical and biomedical applications for the transport and delivery of hydrophobic compounds [17,18,19,20]. Their use is made safe by the low to absent toxicity within definite concentration ranges. 

In order to find the optimal experimental dyeing conditions that guarantee enhanced product functionality and process sustainability at the same time, a preliminary study was performed with quercetin (Figure 1b) solutions at different pH values, since this molecule was found to be the most abundant flavonoid present in the onion skin extract under investigation [12]. The interaction between quercetin and SB3-14, as both a monomer and micellar aggregate, was accessed through spectrophotometric titrations. A series of spectroscopic and chromatographic methods were also applied to evaluate the color, dye uptake, total phenol content (TPC), and total antioxidant capacity (TAC) of wool yarn dyed in the absence and presence of SB3-14 micelles. The toxicity profile of SB3-14 was also deeply investigated.

## 2. Materials and Methods

### 2.1. Chemicals and Reagents

Protocatechuic acid, Brij^®^S10, and 3-(N,N-dimethyltetradecylammonio)propane-1-sulfonate (SB3-14) were purchased from Fluka (Milan, Italy). SB3-14 was purified by twice crystallization from a methanol/acetone mixture. The value of the critical micelle concentration (c.m.c.= 2.88 × 10^−4^ M) was determined from plots of surface tension vs. −log[surfactant] [21]. Quercetin dihydrate (3,3′,4′,5,7-pentahydroxyflavone dihydrate), Folin–Ciocalteu reagent, 2,4,6-tris(2-pyridyl)-s-triazine (TPTZ), 6-hydroxy-2,5,7,8-tetramethyl-2-carboxylic acid (Trolox), 2,2′-azinobis-(3-ethylbenzothiazoline-6-sulphonate) diammonium salts (ABTS), 2,2-diphenyl-1-picrylhydrazyl (DPPH), 2,2′-azobis(2-methylpropionamidine)dihydrochloride (AAPH), hydrochloric acid (HCl), ferric chloride (FeCl_3_), sodium acetate (NaOAc), sodium carbonate (Na_2_CO_3_), sodium chloride (NaCl), potassium persulfate, gallic acid (GA), lactic acid, urea, ammonia solution 32%, phosphate-buffered saline (PBS), fluorescein sodium salt, HPLC grade methanol (MeOH), and ethanol (EtOH) were purchased from Sigma-Aldrich (Milan, Italy). Boric acid (H_3_BO_3_), phosphoric acid (H_3_PO_4_), acetic acid (CH_3_COOH), and sodium hydroxide (NaOH) were from Merck (Milan, Italy). HPLC grade acetonitrile (ACN) and formic acid (HCOOH) were from Sigma-Aldrich (Milan, Italy). Water was purified by using a Milli-Q Plus185 system from Millipore (Milford, MA, USA). PBS solution was prepared as follows: dibasic sodium phosphate (Na_2_HPO_4_, 15.50 g) was dissolved in distilled water (840 mL), and monobasic potassium phosphate (KH_2_PO_4_, 1.52 g) was dissolved in distilled water (160 mL); then, these two solutions were mixed, and pH was fixed at 7.6. 

The study of the interaction between quercetin and the SB3-14 surfactant was carried out in double-distilled water (pH 5.5) and in buffered aqueous solutions in which the pH value (pH 7.0 and 9.0) was achieved by Britton buffers. Britton buffers were prepared by mixing an acidic solution (H_3_BO_3_ 0.04 M, H_3_PO_4_ 0.04 M, and CH_3_COOH 0.04 M) with a 0.2 M solution of NaOH, adjusting the ionic strength of the final solution at 5 × 10^−3^ M. 

Dry onion skins from the “Dorata di Parma” cultivar were kindly provided by the Cannara Onion Producers’ Union (Consorzio dei Produttori della Cipolla di Cannara, Cannara PG, Italy) and processed without further pre-treatments. The raw wool yarn used in this study was a generous gift of a local Umbrian producer.

### 2.2. Photophysical Measurements

Absorption spectra were recorded with a Perkin Elmer Lambda 800 spectrophotometer (PerkinElmer Life and Analytical Sciences, Shelton, CT 06484-4794 USA). The study of the interaction between quercetin and the SB3-14 surfactant was carried out in double-distilled water (pH 5.5) and in buffered aqueous solutions at pH 7.0 and 9.0. Since quercetin is poorly soluble in water, a stock solution (8.9 × 10^−4^ M) was prepared by dissolving the compound in EtOH. Aqueous solutions of quercetin with a concentration of 2.2 × 10^−5^ M were freshly prepared, just before the experiments, by diluting the stock solution of ethanol (50 μL) with 2 mL of water or buffered aqueous solutions. The stock and diluted solutions were stored in the dark and protected from light as much as possible during the experiments. Concentrated solutions of SB3-14 (9.9 × 10^−3^ M) were prepared in pure water and in buffered solutions, at pH 7.0 and 9.0, respectively. Different aliquots of each of these solutions were added to the quercetin solutions to obtain a surfactant concentration ranging from 0 to 1 × 10^−3^ M. Absorption spectra were acquired before and after any surfactant addition. The association constant (K) of the neutral quercetin in water (at pH 5.5) and in monoanionic form (at pH 7.0) to the SB3-14 micelles was determined spectrophotometrically through Equation (1) [22,23]:

(1) $$\frac{1}{A-A_{0}}=\;\frac{1}{\left(A_{\infty }-A_{0}\right)\cdot K\cdot \left(c_{\mathrm{SB}3-14}-\mathrm{cmc}\right)}+\frac{1}{\left(A_{\infty }-A_{0}\right)}$$

where A is the measured absorbance at any surfactant concentration, A_0_ is the initial absorbance (no added surfactant), $A_{\infty }$ is the absorbance value at high SB3-14 concentration, and ($c_{\mathrm{SB}3-14}$ − cmc) is the concentration of micellized surfactant. Folin–Ciocalteau, Ferric Reducing Antioxidant Power (FRAP), ABTS and DPPH tests were carried out at 25 °C with a Thermo Scientific™ Evolution 60 UV-Visible spectrophotometer (Thermo Fisher Scientific, Rodano MI, Italy). Emission intensity for Oxygen Radicals Absorbance Capacity (ORAC) experiments were determined by a HORIBA Scientific FluoroMax®-4P spectrofluorimeter (Kyoto, Japan) operated by FluorEssence™. 

Reflectance spectra on solid samples were performed using a portable instrument composed of Avantes parts and equipped with a quartz fiber optic system already described in a previous paper [24]. The reflectance spectra were expressed in terms of pseudoabsorbance, A′(λ), according to Equation (2) [25]:

(2) $$A^{\prime }\left(\lambda \right)=\log\left[\frac{1}{\left(0.01\cdot R\left(\lambda \right)\right)}\right]$$

where R is the measured reflectance at each specific wavelength λ. Colorimetric measurements on dyed samples (lightness [L*], redness–greenness value [a*], and yellowness–blueness value [b*]) were carried out by light reflectance technique on a Konica Minolta CM-700d spectrophotometer (Tokyo, Japan) under D65 illuminant and 10° standard observer. Four measurements were made for each sample recording the percentage reflectance values over the 350–750 nm spectral range. 

### 2.3. Scouring and Dyeing Procedure

The wool yarn was washed with a Brij^®^S10 non-ionic detergent solution (5 g/L, yarn to liquid ratio 1:100 *w/v*), according to a previously published method [12]. This sample is referred to as untreated wool (UW) along the text.

Wool dyeing experiments were performed following a previously reported procedure [12] with small modifications and halved dyeing times (from 60 to 30 min). Briefly, to dye 2 g of wool, 200 mL of water were added to 2 g of onion skin, and the mixture was kept under magnetic stirring at 95 °C for 1 h. The resulting extract was filtered through paper filter and divided in two baths. One of them was directly used to dye 1 g of wool yarn (Sample T1), whereas the other was added with SB3-14, until a concentration of 2.5 × 10^−3^ M was reached, which was used to dye another sample of wool yarn (Sample T2). For each sample, the dyed wool yarn was carefully rolled around a Plexiglas sheet to have a 3 × 1 cm skein with a flat and homogeneous surface, which was therefore adequate for in situ reflectance and colorimetric measurements.

### 2.4. Preparation of Artificial Sweat 

The artificial sweat was prepared according to the reference test method EN 1811:2011. The solution contained 0.5% (*w/v*) sodium chloride, 0.1% (*w/v*) lactic acid, and 0.1% (*w/v*) urea. The pH was adjusted to 6.5 with ammonia solution.

### 2.5. Treatment of Textiles with Artificial Sweat 

Samples of about 3 mg (on average) of textile dyed in the presence of the zwitterionic surface-active agent were put into a 0.2 mL of artificial sweat (see Section 2.4 for the preparation method) and kept at 37 °C for 2 h. Then, the textile was removed from the solution and allowed to dry at room temperature. An aliquot (0.1 mL) of the resulting artificial sweat solution was submitted to the Folin–Ciocalteu assay, as described in Section 2.6. The same textile was re-immersed in a 0.2 mL (the same volume as before) of artificial sweat and kept at 37 °C for 2 h. The same procedure was repeated for six cycles, for a total of seven determinations. The total phenol content TPC was determined with the Folin–Ciocalteu assay for each of the seven solutions.

### 2.6. Determination of Total Phenol Content (TPC) by the Folin–Ciocalteu Method 

The TPC of each textile was determined in triplicate, according to the Folin–Ciocalteu method described in [26] with only a few modifications. Details on the determination procedure herein applied are reported as Supplementary Materials.

### 2.7. Determination of the Total Antioxidant Capacity (TAC) by the FRAP Method 

The evaluation of the reducing power through the FRAP method was determined according to the procedure already described in [26], with only a few modifications. Details on the determination procedure herein applied are reported as Supplementary Materials.

### 2.8. Determination of the Oxygen Radical Absorbance Capacity by the ORAC Method

The ORAC method was applied by slightly modifying experimental procedures already proposed by other authors [27,28]. Details on the determination procedure herein applied are reported as Supplementary Materials.

### 2.9. Determination of the Radical Scavenging Capacity by the DPPH Method 

The radical scavenging capacity was measured by using the DPPH method, as described in [26]. DPPH was progressively solubilized in HPLC-grade EtOH until a concentration producing an absorbance of 0.65 (±0.02) at 517 nm was reached. Approximately 2 h were required to stabilize the above absorbance value. A volume of 0.05 mL of extract was added to 2.95 mL of DPPH solution. The term “extract” is related to the yarn sample (about 3.0 mg, previously dyed with or without the zwitterionic surfactant) added to a 0.1 mL of water/MeOH (1:1, *v/v*) solution. The absorbance was determined at 517 nm after 30 min of incubation in the dark at room temperature. Analyses were performed in triplicate. Values were determined from a calibration curve prepared with Trolox solutions previously treated by applying the same procedure as for the real sample. Therefore, the antioxidant capacity of the sample was expressed as mg of Trolox equivalents/g textile.

### 2.10. Determination of the Radical Scavenging Capacity by the TEAC/ABTS Method 

The radical scavenging capacity was measured by using the Trolox Equivalent Antioxidant Capacity (TEAC) method as described in [26]. 

A solution of ABTS^+^ was prepared at a concentration of 0.36% (*w/v*) in water, and a solution of potassium persulfate was prepared at a concentration 0.2% (*w/v*) in water. Then, 2 volumes of the ABTS^+^ solution and 1 volume of the potassium persulfate solution were combined. The flask was covered with aluminum foil and allow to stand overnight at room temperature in the dark. The obtained ABTS^•+^ solution was diluted with EtOH until reaching an absorbance of 0.70 (± 0.05) at 734 nm. A 50-fold dilution was required to get the above absorbance value. The analysis was initiated by adding 1.96 mL of ABTS^•+^/EtOH solution to 0.4 mL of extract. The term “extract” is related to the yarn sample (about 3.0 mg, previously dyed with or without the zwitterionic surfactant) added to a 0.1 mL of water/MeOH (1:1, *v/v*) solution. The absorbance was measured at 734 nm after 6 min standing. The analysis was performed in triplicate. Values were determined from a calibration curve prepared with Trolox standard solutions previously treated by applying the same procedure as for the real sample. Therefore, results were expressed as mg of Trolox equivalents/g textile.

### 2.11. HPLC Analysis of Polyphenols in the Extracts

HPLC analyses were performed on an Agilent Technologies (Waldbronn, Germany) modular model 1260 Infinity II system, consisting of a vacuum degasser, a quaternary pump, an autosampler, and a UV/Vis detector. The chromatograms were recorded using an Agilent OpenLab CDS ChemStation Edition (Rev. C.01.10). The analysis of phenolic acids and flavonols was carried out on an Ascentis Express C_18_ column (150 × 3.0 mm I.D., 2.7 µm, Supelco, Bellefonte, PA, USA). The mobile phase was composed of 0.1% HCOOH (*v/v*) in both (A) water and (B) ACN. The gradient elution was modified as follows: 0–5 min 3% B, 5–45 min from 3% to 50% B. The post-running time was 10 min. The flow rate was 0.4 mL/min. The sample injection volume was 10 µL. UV/DAD chromatograms were acquired at 254 nm. Three injections were performed for each sample.

Quantitative analysis was performed by external calibration with the corresponding reference compounds. The stock standard solution of protocatechuic acid and quercetin was prepared by weighing an accurate amount of pure compound and properly diluting it with MeOH. The external standard calibration curve was generated by using five data points, covering the concentration range: 5.5–110.5 µg/mL for protocatechuic acid and 4.5–89.5 µg/mL for quercetin, respectively. Ten µL aliquots of each standard solution were used for HPLC analysis. Injections were performed in triplicate for each concentration level. The calibration curve was obtained by plotting the peak area of the compound at each level versus the concentration of the sample. The amount of polyphenols in onion extracts and residues after dyeing were determined by using the calibration curves of the compounds with the same chromophore. Quantitative data were finally expressed as protocatechuic acid equivalents (μg PAE/mL) and quercetin equivalents (μg QE/mL).

### 2.12. Analysis of Cellular Viability and Apoptosis of SB3-14 on RAW 264.7 Cells

The mouse monocyte/ macrophage cell line RAW 264.7, obtained from the American Type Culture Collection (ATCC, Manassas, VA, USA), was used to investigate SB3-14 cytotoxic and propapoptotic activity. RAW 264.7 cells were cultured according to standard procedures in Roswell Park Memorial Institute 1640 medium (RPMI-1640), supplemented with 10% heat inactivated Fetal Bovine Serum (FBS), 2 mM of L-glutamine, and antibiotics (100 U/mL penicillin, 100 μg/mL streptomycin; Gibco, Invitrogen, Carlsbad, CA, USA). Cells were cultured at 37.0 °C in a 5% CO_2_ for 24 hours in the presence (medium alone) or absence of several concentrations of SB3-14, obtained from a 1 M stock solution in medium RPMI. Cells were resuspended at the concentration of 1.3 × 10^6^ cells/mL in a 12-well plate. The percentage of live, apoptotic, and dead cells was determined by using an Annexin V Apoptosis Detection Kit PerCP-eFluor™ 710 and FVD (Fixable Viability Dye eFluor™ 780; eBioscience, San Diego, CA, USA), according to the manufacturer’s instructions. Specifically, each cell sample was washed and suspended in 100 μL of phosphate-buffered saline before the staining. Then, a dilution 1:1000 of FVD was added to the sample and incubated at 4 °C for 30 min in the dark. Annexin V–PerCP, diluted 1:20, was added and incubated for 15 min at RT in the dark. Flow cytometry analysis was performed within 4 h.

## 3. Results and Discussion

### 3.1. Interaction of Quercetin with SB3-14 Surfactant 

In a previous study [12], quercetin and its glycoside derivatives were found to be the most abundant flavonoids present in water extract of onion skin from the “Dorata di Parma” cultivar. Therefore, this molecule was chosen as a representative species of this class of bioactive compounds to in depth investigate the interaction with SB3-14 zwitterionic surfactant. This preliminary step was necessary to define the optimal experimental conditions in terms of dye uptake and process sustainability. The modeling study of the dyeing step, based on a quantitative spectral chromatographic approach, constitutes an important and essential contribution toward a future standardization process for the production of customized biofunctional textiles.

As typically observed in a flavonoid dye structure, quercetin bears several hydroxyl groups that determine peculiar acid–base properties [29]. Therefore, depending on the pH value of the solution, the molecule is characterized by the predominance of a specific acid–base form and distinctive physical and chemical behavior [30]. Thus, the study of the quercetin–surfactant interaction was carried out at different pH values: double-distilled water (pH = 5.5), which was the medium used to perform dyeing experiments, and pH 7.0 and 9.0 in buffered solutions. According to the literature [29], quercetin is in its neutral form in acidic solutions (pH < 5.0), whereas the monoanionic and bianionic forms are prevalently present at pH 7.0 and 9.0, respectively. Higher pH values were not explored because of the well-known instability of quercetin under extremely alkaline conditions [29]. Moreover, in particularly drastic basic baths, wool structures can undergo serious alteration phenomena, thus compromising the dyeing process [31].

**Figure 2:**
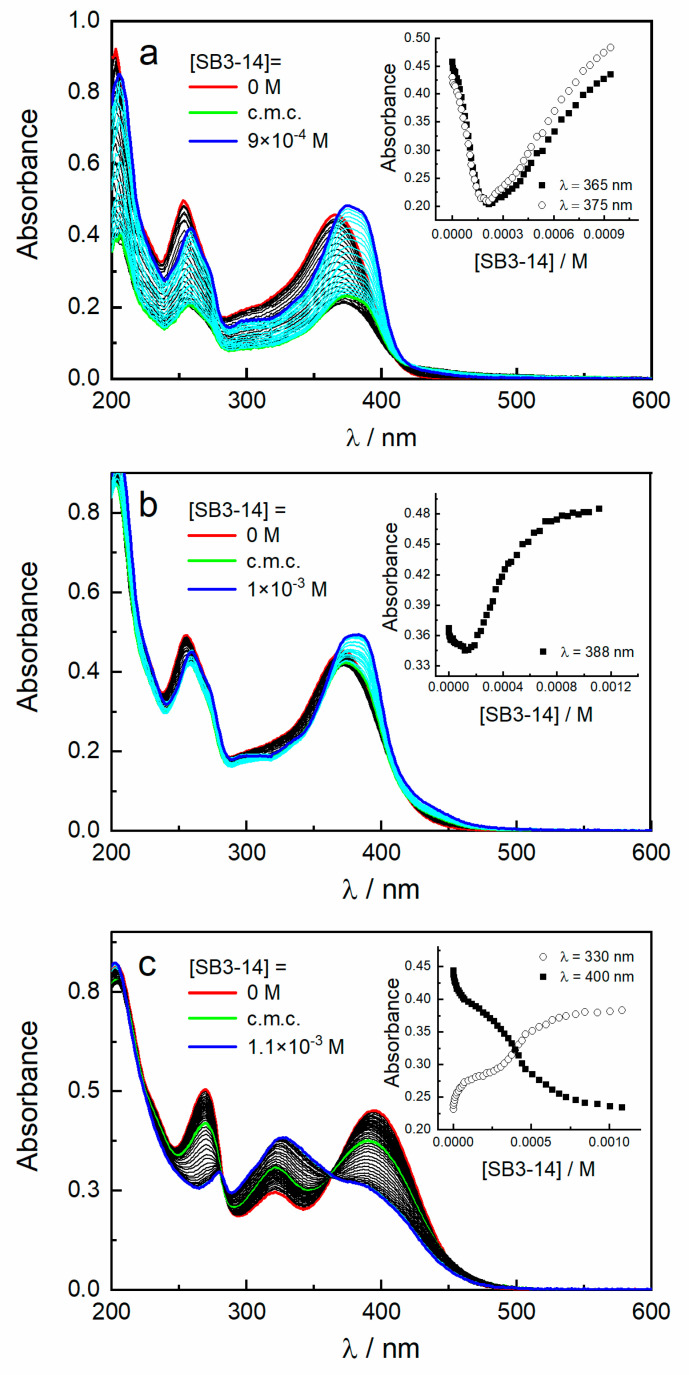
Absorption spectra of quercetin in aqueous pH 5.5 (**a**) and buffered solutions at pH 7.0 (**b**) and 9.0 (**c**) alone and in the presence of increasing amount of SB3-14 up to 1 × 10^−3^ M. Inset: variation of the absorbance of quercetin upon SB3-14 concentration at specific wavelengths.

As far as SB3-14 is concerned, its non-amphoteric nature preserves its chemical alteration regardless of the experimental pH. The interaction between the different prototropic species of quercetin and SB3-14 was studied by spectrophotometric titrations performed by adding increasing amounts of surfactant to an aqueous solution of the dye, as displayed in Figure 2. In double-distilled water, the absorption spectrum of the neutral form of quercetin is characterized by a band with maximum at 366 nm (band I) and a band centered at 253 nm (band II) with a weak shoulder at about 270 nm (Figure 2a). Gradual additions of the surfactant, at concentrations below its critical micellization concentration (c.m.c.), induce a sharp decrease of absorbance together with a slight bathochromic shift of both bands I and II. The presence of aromatic rings in the quercetin structure and the absence of neat charges make the molecule poorly soluble in water; therefore, an addition of surfactant monomers determines a “salting out” effect that decreases the solubility of quercetin and consequently its absorbance. With further addition of SB3-14 above the c.m.c., an increase of absorbance of both bands I and II is detected, and the formation of a new shoulder at 388 nm is observed, clearly indicating an interaction between quercetin and surfactant aggregates. The double absorption trend just described, and well evidenced in the insert of Figure 2a, is typical of lipophilic solutes interacting with surfactant molecules entering the hydrophobic micellar core [22,32]. A high binding constant (K) value of 800 M^−1^ is obtained, implying a great affinity of the neutral form of quercetin with SB3-14 micelles. The retrieved value is of the same order of magnitude of that found for an association of quercetin, in solutions with similar concentration, with anionic sodium dodecyl sulfate (SDS) and sodium bis(2-ethylhexyl)sulfosuccinate (AOT) surfactant micelles [33,34].

In buffered solution at pH 7.0, quercetin is mainly present as a monoanion in equilibrium with small amounts of neutral and bianionic forms. The spectral modifications induced by the addition of increasing amount of SB3-14 reflect the double trend already observed in water but with drastically reduced absorbance variations (Figure 2b). This feature implies a stronger interplay between the monoanionic form of quercetin with both monomer and micelle surfactants because of hydrophobic and electrostatic forces, as confirmed by the higher K value (7600 M^−1^).

Instead, a peculiar behavior is observed for the bianionic form of quercetin, as displayed in Figure 2c. Indeed, the addition of surfactant leads to a progressive reduction of the absorbance of both band I (at 395 nm) and II (at 270 nm), with a concomitant increase of intensity for the signal at 320 nm. The presence of two isosbestic points (at 280 and 363 nm) highlights an equilibrium between quercetin and a decomposition product. An analogue spectral evolution has been reported for quercetin in water at pH 7.0 [35] and in nitrogen atmosphere Britton buffers at pH > 9.0 [29] and has been assigned to the formation of an oxidation product [36]. 

**Figure 3:**
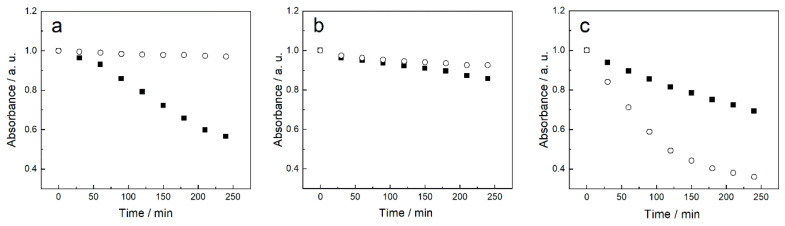
Comparison of time variation of the absorbance at specific wavelengths, referred to its t = 0 value, of quercetin (5 × 10^−5^ M) aqueous solutions in the absence (filled squares) and presence (open circles) of SB3-14 surfactant micelles (2.5 × 10^−3^ M) in double-distilled water at pH 5.5 at 373 nm (**a**), pH 7.0 at 370 nm (**b**), and pH 9.0 at 395 nm (**c**).

The role played by SB3-14 surfactant micelles on the stability of the various acid–base forms of quercetin has been evaluated by recording the absorbance as a function of elapsed time at specific wavelengths, depending on the pH (double-distilled water at 373 nm, pH 7.0 at 370 nm, and pH 9.0 at 395 nm). As displayed in Figure 3, in the absence of surfactant, the absorbance of quercetin decreases with time, referred to its value at t = 0, thereby indicating a certain instability that occurs with a greater extent in double-distilled water. The presence of surfactant micelles results in a stabilizing effect that is more remarkable in water than pH 7.0 (Figure 3a,b). On the contrary, at pH 9.0, the micelles notably accelerate quercetin degradation (Figure 3c). Therefore, this pH value was discarded for dyeing experiments.

Based on the results obtained from the preliminary study on the surfactant–quercetin interaction, the wool yarn was dyed using double-distilled water as a dye bath and SB3-14 in a concentration higher than the c.m.c. (2.5 × 10^−3^ M). These experimental conditions represent the best compromise between quercetin stabilization and process sustainability. Indeed, the simple use of SB3-14 micelles in water not only prevents quercetin from degradation but also reduces the use of further chemical reagents necessary for pH control of the dye bath. 

### 3.2. Effect of SB3-14 on Dyed Wool Properties

**Figure 4:**
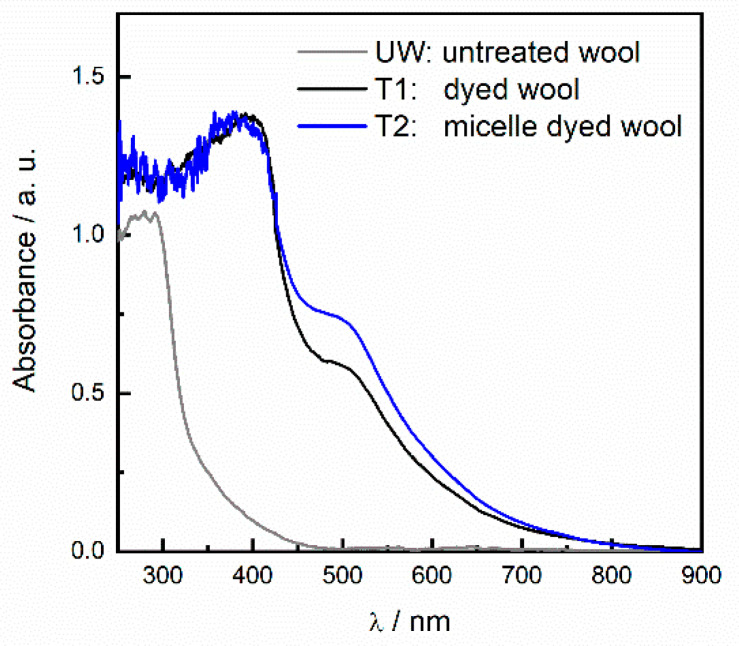
Absorption spectra of untreated wool (UW, gray) and dyed wool in aqueous onion skin extract in the absence (black) and presence (blue) of SB3-14 micelles.

The absorption profile of textile samples T1 and T2 (Figure 4) shows the characteristic flavonoid absorption band I, centered at 390 nm, and a bathochromic unidentified feature at 510 nm, probably due to flavonoid aggregates [12], resulting in a deep red-brown color as reported in Table 1. The higher absorbance values detected in the visible spectral range (Figure 4) for the sample dyed in the presence of micelle surfactant (T2) clearly indicate a greater dye uptake. As already reported [12], the spectra of the textile samples show absorption values higher than the untreated wool in all the UV-Vis spectral range including the UVa and UVb, highlighting a certain potential protection function from UV radiations on human skin. 

In order to evaluate the total phenol content (TPC) released from the treated wool samples, as well as the corresponding total antioxidant activity (TAC), a number of spectroscopic analyses were performed. Accordingly, TPC was determined spectrophotometrically by applying the original Folin–Ciocalteu method with only a few modifications, as described in our previous work [12,26]. The amount of dyed wool spanning the range from 2.5 to 4.9 mg was used for the TPC analyses. The same samples were also used to appraise the TAC of the produced biofunctional textiles. Accordingly, three different spectrophotometric assays (FRAP, DPPH, and ABTS) and a spectrofluorimetric assay (ORAC) were performed. The FRAP method is exclusively based on a single electron transfer (ET) mechanism, while ORAC can be defined as a “pure” hydrogen atom transfer (HAT) process that measures the ability of an antioxidant (or a mixture of antioxidants) to quench free radicals through hydrogen donation. In the present study, DPPH and ABTS assays were also performed, whose radicals are recognized to activate mixed-mode (HAT and ET) reactions depending on the applied experimental conditions [37,38].

The results of the above tests are summarized in Table 2. A slight but consistent increase in the TPC and TAC values were always measured for solutions containing wool samples dyed in the presence of SB3-14, irrespective of the test considered. These findings are in strict accordance with the spectral data shown in Figure 4, indicating that the presence of SB3-14 micelles in the dye bath favors the dye uptake on wool fibers. This evidence suggests that the use of the zwitterionic surfactant as a component of the dying bath can be a safe (see Section 3.4 for details), feasible, and potentially scalable strategy to improve the biofunctional properties of wool-based textiles. However, it must be pointed out that, in order to gain more sizeable advantages from the dying process in the presence of the surface-active agent, a further optimization of the production process is strongly required. In this context, methodological improvements enabling a more homogeneous penetration of the vehiculated molecules within the textile fibers (which means narrower standard deviation ranges) are necessary. Accordingly, the comparative screening of different zwitterionic compounds could be useful to this aim, along with the use of a non-toxic natural mordants in the bath solution that could synergistically operate with it. 

**Figure 5:**
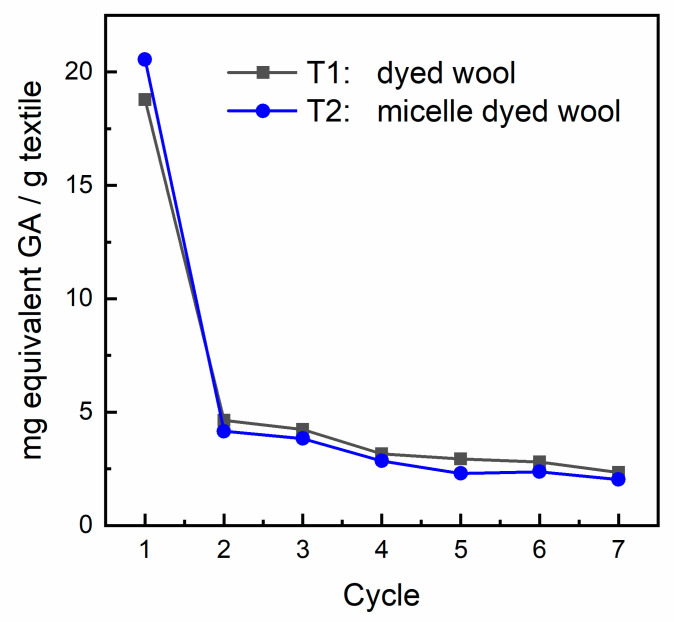
Release of the phenolic pool from the textile in an artificial sweat solution.

In order to virtually evaluate the release of the phenolic pool from the wool matrix, samples of textiles dyed in the presence of the zwitterionic surfactant were treated with artificial sweat (see Section 2.4 and Section 2.5 for details). An aliquot of the resulting solution was submitted to the Folin–Ciocalteu assay as described in Section 2.6. About 3 mg (on average) of treated textiles were submitted to 7 cycles of the Folin–Ciocalteu assay. The results expressed as mg equivalents GA/g textile are shown in Figure 5. The results of this study clearly indicate that the phenolic pool is rapidly delivered from the fiber, with a higher release from the sample treated with the surface-active agent. A plateau is reached soon after the first “dry-and-soak” cycle with a minor but constant release of phenols from both types of sample. A visual inspection of the fiber repeatedly submitted to the analysis did not reveal any substantial change in color intensity; thus, our original idea that only a minor amount of superficially adsorbed polyphenols is responsible for the previously observed biofunctional properties is confirmed.

### 3.3. Quantitative Analysis of Phenolic Acids and Flavonols in the Extracts

**Figure 6:**
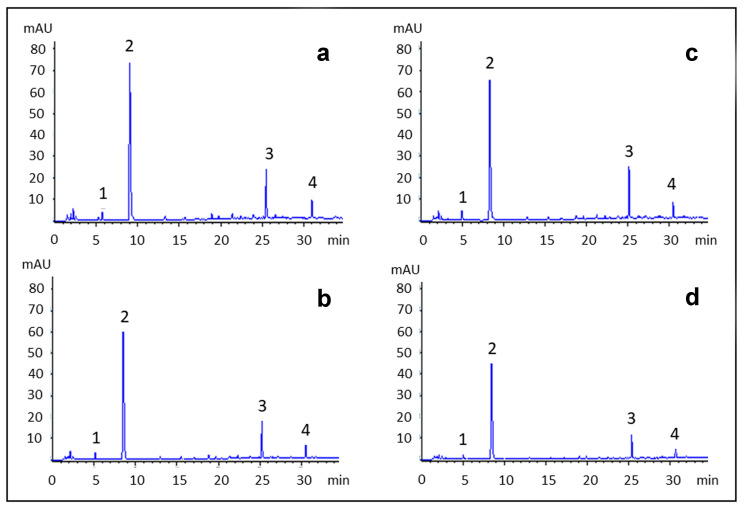
HPLC-UV chromatograms of onion extracts (**a**,**c**) and the residues after the dyeing process (**b**,**d**) in the absence (**a**,**b**) and in the presence (**c**,**d**) of the surfactant, acquired at 254 nm. Peak identification: 1. protocatechuic acid-glucoside, 2. protocatechuic acid, 3. quercetin-3-*O*-glucoside, 4. quercetin.

In order to further demonstrate the higher uptake of polyphenols promoted by the employed surfactant, a quantitative HPLC-UV analysis of phenolic acids and flavonols in the extract was performed. Indeed, the onion extracts considered in this study were analyzed by means of a HPLC-UV method previously optimized in order to quantify the main polyphenols [12]. Representative HPLC-UV chromatograms of the extracts both before and after the dyeing process both in the presence (ET2) and absence (ET1) of the surfactant are shown in Figure 6. From a qualitative point of view, onion extracts contained almost exclusively phenolic acids and flavonols: in particular, protocatechuic acid and quercetin-3-O-glucoside were the most abundant compounds found in all the extracts analyzed, followed by quercetin and protocatechuic acid–glucoside (Figure 6). The identification of these compounds was performed with reference standards, UV, MS and MS^2^ data, as previously described [12].

Looking at the quantitative data shown in Table 3, a little difference was observed in the content of both chemical classes of compounds between the onion extract and its residue after the dyeing process in the absence of the surfactant (from 100.0 to 91.8 µg/mL for PAE and from 26.2 to 22.0 µg/mL for QE, respectively), suggesting a limited adhesion of onion polyphenols to the fiber. On the other hand, the addition of the surfactant improves the transfer of onion polyphenols from the extract to the fiber, as their amounts in the residue after the dyeing procedure decreased significantly (from 109.1 to 56.0 µg/mL for PAE and from 32.4 to 15.7 µg/mL for QE, respectively). 

### 3.4. Determination of SB3-14 Cytotoxicity on RAW 264.7 Cells

To evaluate the potential cytotoxic effect of SB3-14 in both monomeric and aggregated forms, cell viability and induction of apoptosis were evaluated in RAW 264.7 cells, which is a murine monocyte/macrophage cell line generally used to assess the toxicity of biologically active compounds and/or their effects on inflammatory processes and immune responses.

**Figure 7:**
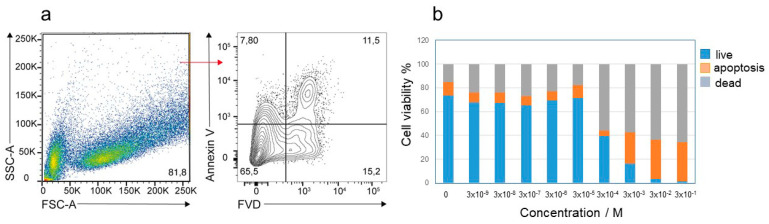
(**a**) RAW264.7 cells were left untreated or treated for 24 h with SB3-14 at the indicated concentrations. Cells were stained using the PerCP–Annexin V and FVD 780 and analyzed by flow cytometry. Annexin V/FVD—double negative cells (lower left quadrant) represented live cells, annexin V/FVD—double positive cells (upper left and upper right quadrant) represented apoptotic cells, and annexin V-negative/FVD-positive cells (lower right quadrant) indicated dead cells. A representative dot plot (relative to untreated cells) is shown. The percentage of viable, apoptotic, and dead cells was reported in (**b**) for each SB3-14 concentration. Data are the mean percentage of two different experiments.

RAW264.7 were either untreated or treated by using nine tenfold dilutions of SB3-14 in the range between 3 × 10^−9^ and 3 × 10^−1^ M. After 24 h of incubation, cell viability and apoptosis were evaluated by means of a cytofluorimetric analysis (Figure 7a). As shown in Figure 7b, cell viability drastically decreased below 50% at concentrations of SB3-14 above 3 × 10^−4^ M, suggesting a cytotoxic effect. It is interesting to notice that 3 × 10^−4^ M is very close to the c.m.c., whereas at concentrations under the c.m.c., the surfactant shows no or very low cytotoxic effects. 

Figure 7b also shows that the cytotoxic effect at concentrations of SB3-14 higher than 3 × 10**^−4^** M is accompanied by an increase in the proapoptotic effect. Conversely, the overall percentage of viable and apoptotic cells in samples treated with concentration of SB3-14 lower than 3 × 10^−5^ M is comparable to that of untreated cells.

These analyses revealed that SB3-14 is safe for the RAW264.7 cell line in a concentration range under the c.m.c., which is in its unaggregated form. 

## 4. Conclusions

We have recently demonstrated that the onion skin from the “Dorata di Parma” cultivar can be used as a source of biomolecules for the sustainable production of biofunctional textiles with antioxidant and UV-protective properties [12]. The present paper intends to describe a further advancement of this research, by focusing on the addition of the SB3-14 zwitterionic surfactant in the dyeing bath. In the present study, we have clearly demonstrated that the use of SB3-14 micelles markedly improves the dye uptake on the wool yarn, with a potential profitable effect on the biofunctional properties of the modified textile (antioxidant/antiradical profile). In order to improve the dyeing process, a preliminary physicochemical investigation was performed with quercetin as the model compound, since this molecule has already been identified as the most abundant one in the extracted pool of phenolics from onion skin. This step was found to be fundamental, since it allowed us to gain important qualitative and quantitative information for an optimal definition of the best experimental conditions for the dyeing step. It was highlighted that, depending on the pH of the solution, the surfactant concentration differently influences both the dye solubility and stability. In particular, when the surfactant is present as a monomer (at concentrations below its c.m.c.), quercetin solubility decreases, due to salting out effects. Instead, in the presence of micelles (at concentration above its c.m.c.), the dye solubilization is efficiently improved, especially in water (pH 5.5). On the other hand, the affinity of quercetin for SB3-14 micelles was stronger at pH 7.0 than in water, as demonstrated by the K value that at the latter pH is one order of magnitude higher (7600 M^−1^) than in water (800 M^−1^). However, a strong dye–surfactant interaction is not always desirable in dyeing textiles, due to the possible competition with the attractive forces between the dye and the fiber, thus compromising the absorption of the dye into the fabric substrate. A peculiar behavior was observed at pH 9.0, where the addition of the surfactant significantly accelerates the well-known instability of quercetin; therefore, this pH was not taken into account for the dyeing experiments. Instead, an opposite trend was observed at pH 5.5 and 7.0, where the presence of micelles reduced or even prevented quercetin from its natural degradation. For the aforementioned reasons, the dyeing of wool with aqueous extract of dry onion skin was carried out in water at pH 5.5 in the presence of SB3-14 micelles. 

A cytotoxicity study revealed that SB3-14 surfactant is toxic for the RAW264.7 cell line at concentrations higher than its c.m.c. value. However, considering the abundant washing cycles following the dyeing step, the concentration of the surfactant on wool fibers and waste waters is rationally and realistically expected to be extremely low—that is, well below its c.m.c.—and therefore in a concentration range where the unaggregated form of the surfactant was found to be not toxic. 

The introduction of the surfactant in the dyeing bath (pH = 5.5) at a concentration higher than its c.m.c. value leads to an increase in dye uptake on wool fibers, as demonstrated by the higher absorbance values and total phenol content (measured with the Folin–Ciocalteu assay), resulting in higher antioxidant activity (measured with the FRAP, DPPH, ABTS, and ORAC assays). The higher loading of polyphenols on the wool fibers in the presence of SB3-14 surfactant was further confirmed by quantitative HPLC-UV analysis. It is interesting to notice that, with respect to previously reported experiments [12], the dyeing time in the presence of SB3-14 was approximately halved, with a considerable gain in terms of energy and time consumption.

This study opens new perspectives in the use of surfactants for the production of biofunctional fabrics from biomasses, meeting the ecological fundamentals of the circular economy paradigm. The experimental outcomes of the present study indicated that the use of the selected zwitterionic surfactant in the dyeing bath can enter the frame of an efficient and safe strategy to ameliorate the biofunctional properties of wool-based textiles. However, an optimization study is still required to further improve the production process, by exploring the effect of different zwitterionic compounds, along with the use of non-toxic natural mordants in the bath solution for a synergistic action. Moreover, besides the commonly known leveling, dispersing, and wetting properties, many surfactants bear their self-functional properties (antibacterial, antifungal, etc.) or they can confer peculiar behavior when they are adsorbed on textile surface (softener, antistatic, water proof activity, just to mention a few). Therefore, the combined use of natural flavonoid dyes and surfactants can represent a valuable strategy for the sustainable and functional finishing of textiles as well.

## Figures and Tables

**Table 1:** Colorimetric coordinates of textile samples.

Sample	L*	a*	b*
**T1**	55.44	13.48	25.53
**T2**	50.24	15.26	23.39

lightness [L*], redness–greenness value [a*], and yellowness–blueness value [b*].

**Table 2:** Summary of the Folin–Ciocalteu, FRAP, 2,2-diphenyl-1-picrylhydrazyl (DPPH), 2,2′-azinobis-(3-ethylbenzothiazoline-6-sulphonate) diammonium salts (ABTS), and ORAC values measured on the textile extracts.

Sample	Type of Assay				
	FOLIN–CIOCALTEU	FRAP	DPPH	ABTS	ORAC
	mg Eq GA/g Textile	mg Eq TROLOX/g Textile			
**T1**	17.29 ± 0.67	5.98 ± 0.50	2.14 ± 1.16	4.17 ± 2.15	9.38 ± 1.72
**T2**	21.82 ± 2.77	7.97 ± 1.70	4.62 ± 1.66	4.34 ± 0.24	11.41 ± 1.54

**Table 3:** Quantitative analysis of protocatechuic acid, quercetin, and their glucosides in onion extracts and their residue after dyeing procedures. Data are expressed as mean (*n* = 3) ± SD.

Sample	Protocatechuic acid and Derivatives (µg PAE/mL)	Quercetin and Derivatives (µg QE/mL)
**ET1**	100.0 ± 11.0	26.2 ± 3.1
**ET1-post**	91.8 ± 2.7	22.0 ± 0.1
**ET2**	109.1 ± 4.3	32.4 ± 3.5
**ET2-post**	26.2 ± 3.1	15.7 ± 1.4

## Supplementary Materials

The following are available online at https://www.mdpi.com/2076-3921/9/11/1055/s1, mination of total phenol content (TPC) by the Folin-Ciocalteu method; Determination of the total antioxidant capacity (TAC) by the FRAP method; Determination of the oxygen radical absorbance capacity by the ORAC method.
