# Supplementary material for: Use of a Zwitterionic Surfactant to Improve the Biofunctional Properties of Wool Dyed with an Onion (Allium cepa L.) Skin Extract
Source: Antioxidants (Basel). 2020 Oct 28;9(11):1055. doi: 10.3390/antiox9111055 (PMC7693141; doi:10.3390/antiox9111055)
Supplement: Supplementary file 1 [file antioxidants-09-01055-s001.pdf]

Supplementary Materials

Use of a zwitterionic surfactant to improve the biofunctional properties of wool dyed with an onion (*Allium cepa* L.) skin extract

Chiara Puri [1], Lucia Pucciarini [2], Matteo Tiecco [1], Virginia Brighenti [3], Claudia Volpi [4], Marco Gargaro [4], Raimondo Germani [1], Federica Pellati [3], Roccaldo Sardella *[2,5] and Catia Clementi *[1]

[1] Department of Chemistry Biology and Biotechnology, University of Perugia, Via Elce di Sotto 8, 06123 Perugia (Italy); puric.biotec@gmail.com; matteotiecco@gmail.com; raimondo.germani@unipg.it; catia.clementi@unipg.it
[2] Department of Pharmaceutical Sciences, University of Perugia, Via Fabretti 48, 06123 Perugia (Italy); lucia.pucciarini@hotmail.it; roccaldo.sardella@unipg.it
[3] Department of Life Sciences, University of Modena and Reggio Emilia, Via G. Campi 103, 41125 Modena (Italy); virginia.brighenti@unimore.it; federica.pellati@unimore.it
[4] Department of Experimental Medicine, University of Perugia, P.le Severi, 06132 Perugia (Italy); claudia.volpi@unipg.it; marco.gargaro@unipg.it
[5] Center for Perinatal and Reproductive Medicine, University of Perugia, Santa Maria della Misericordia University Hospital, 06132 Perugia (Italy); roccaldo.sardella@unipg.it
* Correspondence: roccaldo.sardella@unipg.it (R.S); catia.clementi@unipg.it (C.C)

mination of total phenol content (TPC) by the Folin-Ciocalteu method

The Folin-Ciocalteu reagent was diluted 10-fold with water. A definite volume of extract (0.1 mL) was mixed with 0.75 mL of the diluted Folin-Ciocalteu reagent and incubated in the dark for 10 min at room temperature. The term "extract" is related either to the yarn sample (about 3.0 mg, previously dyed with or without the zwitterionic surfactant) added to a 0.1 mL of water/MeOH (1:1, v/v) solution or to the textile extract obtained with artificial sweat as described in section 2.4. Then, 0.75 mL of 2% Na_2CO_3 (w/v) aqueous solution were added. The mixture was kept in the dark for 3 h before measuring the absorbance at 765 nm. The content of total phenolics was determined by using a standard curve prepared with gallic acid (GA) solutions previously treated in the same way as for the real samples. Therefore, results were expressed as mg of GA equivalents/g textile.

Determination of the total antioxidant capacity (TAC) by the FRAP method

The FRAP reagent was prepared from 2.5 mL of a TPTZ solution (10 mM) in HCl (40 mM) and 2.5 mL of a $FeCl_3$ aqueous solution (20 mM), mixed with 25 mL of NaOAc (300 mM, pH 3.6). For the determination of the antioxidant activity, 1.5 mL of FRAP reagent were mixed with 0.1 mL of the extract. The term "extract" is related to the yarn sample (about 3.0 mg, previously dyed with or without the zwitterionic surfactant) added to a 0.1 mL of water/MeOH (1:1, v/v) solution. The reaction mixture was allowed to stand for 4 min at room temperature before measuring the absorbance at 593 nm. Analyses were performed in triplicate for each sample. The total antioxidant capacity (TAC) values were determined from a calibration curve prepared with Trolox standard solutions, previously treated by applying the same procedure as for the real sample. Therefore, the antioxidant capacity of the sample was expressed as mg of Trolox equivalents/g textile.

Determination of the oxygen radical absorbance capacity by the ORAC method

A fluorescein solution (42 nM in PBS) freshly prepared each day was pre-incubated in a warm water bath (37 °C) for at least 15 min before measurements. AAPH (153 mM) was prepared daily by solubilizing 400 mg of AAPH in 10 mL PBS. In each cuvette, 2.25 mL of fluorescein and either 0.375 mL of extract (the term "extract"

is related to about 2 mg of yarn sample previously dyed with or without zwitterionic surfactant added to a 5 mL of PBS), or the same volume of blank (PBS), or of the standard solution (Trolox, 6, 12.5, 25 and 50 μM) were placed. Then, 0.375 mL of AAPH were added. The fluorescence was measured immediately after the AAPH addition and measurements were then made every 5 min until the relative fluorescence intensity was less than 5% of the value of the initial reading. The measurements were made in triplicate. Analysis were carried out on a spectrofluorimeter by fixing the excitation and emission wavelength at 493 nm and 515 nm, respectively. Both excitation and emission slits were set at 2.5 nm.

The ORAC final calculations were made by applying a regression equation built up with trolox (used as standard) concentrations (x-axis) vs AUC (y-axis) values. AUC represents the area under the fluorescence decay curve, calculated as follows:

$$AUC = \left[5 + \frac{f5}{f0} + \frac{f10}{f0} + \frac{f15}{f0} + \cdots\right] * 5$$

Where f0 is the initial fluorescence intensity before AAPH addition at time 0, and f5, f10, f15 are the fluorescence intensity at time 5, 10, 15 minutes after the addition of AAPH.

All the obtained AUC values were subtracted of that found blank. Results were expressed as mg of Trolox equivalents/g textile.
